# Experimental investigation of binder based on rice husk ash and eggshell lime on soil stabilization under acidic attack

**DOI:** 10.1038/s41598-022-11529-6

**Published:** 2022-05-09

**Authors:** Joice Batista Reis, Giovana Pelisser, William Mateus Kubiaki Levandoski, Suéllen Tonatto Ferrazzo, Jonas Duarte Mota, Adriana Augustin Silveira, Eduardo Pavan Korf

**Affiliations:** 1 Environmental and Sanitary Engineering, Universidade Federal da Fronteira Sul - Campus Erechim, Erechim, RS 99700-970 Brazil; 2 Graduate Program in Environmental Science and Technology, Universidade Federal da Fronteira Sul - Campus Erechim, Erechim, RS 99700-970 Brazil; 3 Graduate Program in Civil Engineering, Universidade Federal do Rio Grande do Sul, Porto Alegre, RS 90035-190 Brazil; 4 College of Engineering and Architecture, Universidade de Passo Fundo, Passo Fundo, RS 99052-900 Brazil

**Keywords:** Engineering, Civil engineering, Environmental sciences

## Abstract

This study evaluates the use of rice husk ash (RHA)-eggshell lime (ESL) and RHA-commercial lime (CL) as alternative binders for clayey soil stabilization, as well as the performance of soil-binder mixtures under acidic attack. A central composite design was carried out to analyze the reactivity by batch tests with a sulfuric acid solution. Physical and mechanical behavior was evaluated by compaction test and unconfined compressive strength (UCS). Reactivity tests demonstrated better neutralization of contaminant acidity for mixtures with ESL. The highest compressive strength, reactivity and partial encapsulation of toxic elements are associated with application of 30% RHA and 6% ESL in the soil. A C–S–H gel is observed in poorly crystalline phases through the XRD pattern. The application of RHA-ESL in soils exposed to acidic attack has environmental feasibility. Analysis of RHA grinding processes combined with the mixture strength over time, and its application tests in impermeable barriers, in landfills, are recommended.

## Introduction

Solid waste represents a growing concern at the global level, mainly due to population growth and consumerism^1^. A major waste generator is the world’s production of rice, estimated to produce 750 million tons of rice husk yearly^2^. Furthermore, there is a new field of studies related to the valuation of waste from the food sector, the eggshell. The world generates 4.91 million tons of eggshell waste annually, being 278,250 tons in Brazil^3^.

Rice processing generates 160 million tons of waste composed of rice husk, which is destined to landfills^2^ or for energy purposes^4^. The portion of rice husk used for energy purposes is regularly used as fuel in boilers for energy production, and after burning, it generates a new waste: rice husk ash (RHA). RHA is considered a pozzolana due to its source of amorphous silica^4^; which promotes the application of RHA in multiple destinations, such as production of concrete^5–7^, mortars^8,9^, soil remediation processes^10^, adsorption^11^; constitution of geopolymers^12^, and effluent treatment^13^.

The characteristics of RHA and its potential as a pozzolanic material strongly depend on the methods and conditions that originated the RHA^4,14^. RHA from different industries may have different amorphous silica content and specific surface areas, as well as other distinct characteristics that are still poorly explored^14,15^. Therefore, not only the characterization of RHA is important, but also the exploration of its use in mixtures with materials that increase the alkalinity of the environment (e.g. lime).

In this context, many studies have evaluated the use of eggshell waste as an alternative calcium source for different geotechnical and civil construction applications^3,16–20^. Furthermore, studies observed that eggshell lime (ESL) presents itself as an excellent material for soil stabilization along with a pozzolanic source, such as RHA, and a more sustainable binder than dolomitic lime because it avoids limestone mineral extraction and improvement^4,20,21^.

Although these studies explore the isolated application of RHA and eggshell lime for the development of new materials, there is a lack of studies using these two materials in a mixture to improve the mechanical and reactive behavior of compacted clayey residual soils. These mixtures can be applied to waterproofing barriers in landfills, for example, and subject to contact with acidic contaminants from the disposal of waste or tailings. These barriers can prevent degradation in its microstructure and prevent the migration of contaminants to the subsoil^22^.

This paper fills this gap by comparing RHA-eggshell lime with RHA-commercial lime as alternative binders for soil stabilization. An experimental design was performed to investigate factors that could significantly influence the reactivity of clayey soil-RHA-lime mixtures. In addition, characterization of rice husk ash, unconfined compressive strength test, environmental performance, chemical and mineralogical analyses were conducted in mixtures.

## Materials and methods

**Figure 1:**
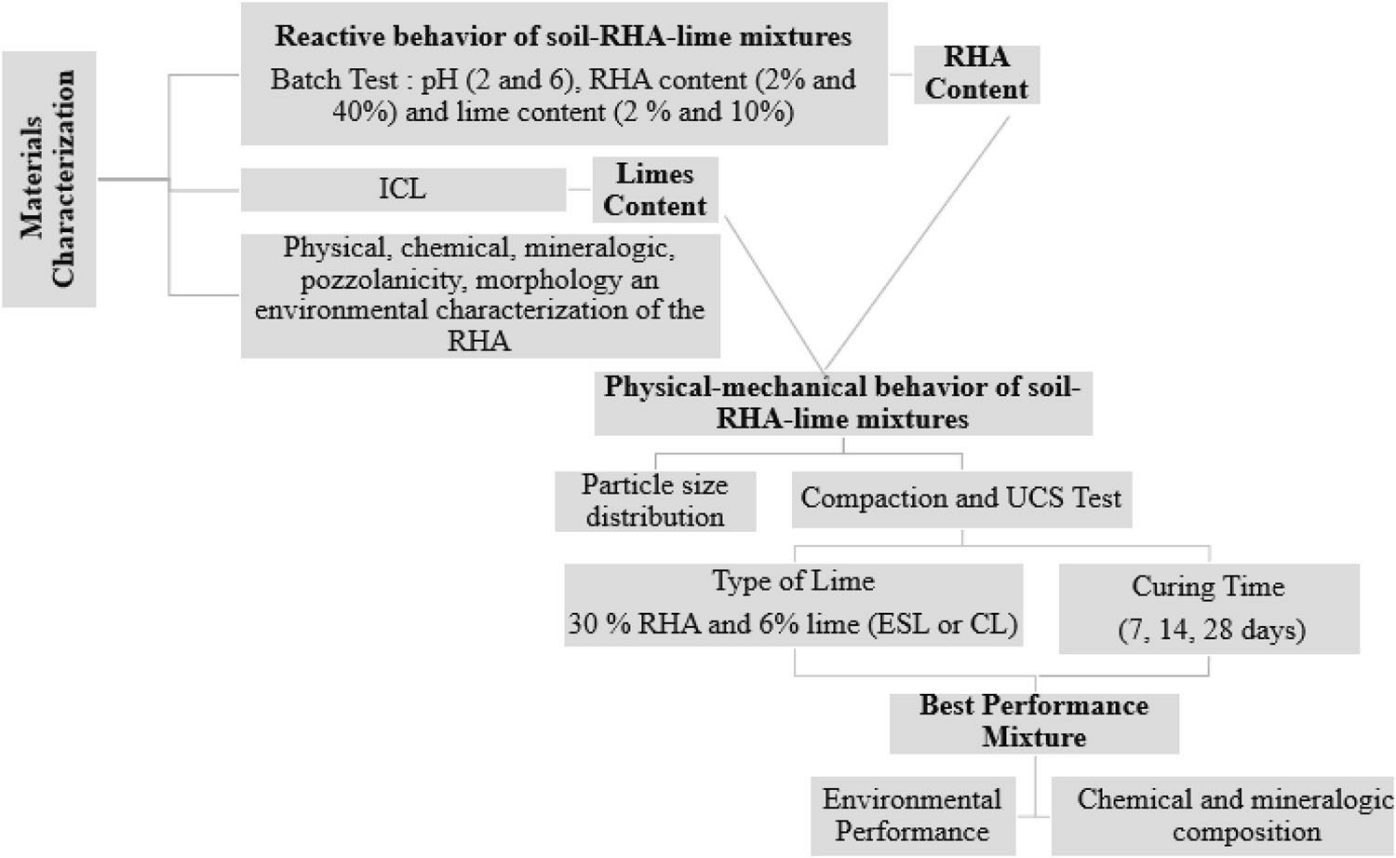
Study methodology flowchart.

Figure 1 presents the materials and methods used in this study, including: materials characterization; tests to determine the composition of the alternative binder; and physical–mechanical, environmental, chemical and mineralogical analysis of soil-binder mixtures.

### Materials

Materials used were residual basalt soil, rice husk ash (RHA), eggshell lime (ESL), and hydrated commercial lime (CL). The residual basalt soil used was collected in the experimental area of the Federal University of Fronteira Sul, Brazil. The RHA comes from a thermoelectric plant located in the state of Rio Grande do Sul, Brazil, that burns the rice husk at a temperature of 800–1000 °C for 8–12 s, and then slowly cooling to room temperature. The RHA was used without any prior processing.

Eggshell lime (ESL) was produced in laboratory^23^. First, the eggshells were washed with distilled water and subjected to a drying process in an oven at a temperature of 105 °C for 24 h, and then they were ground in a knife mill and calcined at a temperature of 1200 °C for 6 h. For comparison purposes, a hydrated commercial lime (dolomitic lime) was used because it is frequently applied for soil improvement in Brazil^4^.

### Materials characterization methods

The RHA was collected at the thermoelectric plant and prepared according to the Brazilian standard NBR 10007^24^. The parameters of moisture content and pH were determined according to Brazil^25^. The physical characterization of the material was performed by testing the specific gravity of the sample, following D854 standard^26^. The determination of the specific surface area of the RHA was performed by analyzing Brunauer, Emmett and Teller—BET isotherms, using nitrogen. In addition, the size of the particles was characterized both through laser, using the laser diffraction equipment and particle analyzer, model Cilas-1064.

The RHA was further chemically characterized by X-ray fluorescence spectrophotometry (XRF) analysis, using a Malvern Panalytical^®^ X-ray fluorescence spectrometer, Zetium model with STD-1 (Standardless) calibration. The samples were pressed for analysis, performed with patterns of chemical elements between fluorine and uranium. The quantification of the organic matter content in the RHA was carried out employing a quali-quantitative determination of the material's loss on ignition. The firing conditions were at a temperature of 1020 °C for 2 h, with detection of 0.1% and normalization at 100%.

The mineralogical composition of rice husk ash was analyzed through X-ray diffraction (XRD) analysis by the powder method. The analysis was performed using a copper tube with voltage parameters of 45–40 kV/mA, angular variation of 2°–70°, with a step of 0.02° every 300 s. The pozzolanicity of RHA was qualitatively evaluated, according to the Fratini chemical test, according to the European standard^27^ and mineralogical composition.

To study the morphology and microstructure of the particles, the samples were analyzed using scanning electron microscopy (SEM), Tescan brand equipment, model Vega 3. The following analytical conditions were used in the analyses: backscattered electrons (BSE) with magnification of 40, 150, 500, and 3000 times, electron beam of voltage of 10 kV.

Additionally, the RHA was characterized as to leaching and solubilization of contaminants for environmental classification, according to Brazilian standards NBR 10004^28^, NBR 10005^29^, and NBR 10006^30^. The extracts had their chemical composition analyzed and compared with the limits imposed in Annex F for leachate and Annex G for solubilized, both present in NBR 10004^28^.

The soil under study was characterized in terms of its particle size distribution, according to^31^, as well as for the test of specific unit weight of grains^26^.

### Material properties

Table 1 presents physical properties of RHA. The RHA is composed of low humidity and alkaline pH (9.50).

**Table 1:** Physico-chemical characterization of RHA.

Properties	RHA
pH	9.50
Water content (%)	< 1
Specific unit weight of grains (g/cm^3^)	2.17
Specific surface area (m^2^/g)—BET	11.02

As noted, the RHA has a low specific unit weight of grains (Table 1) when compared to the soil used (2.58 g/cm^3^). Kumar and Gupta^32^ observed a similar specific unit weight of grains of the RHA, with a value of 1.98 g/cm^3^. The incorporation of RHA in the soil leads to a decrease in its density, as observed by Qu et al.^33^. However, based on the results, rice husk ash can increase soil quality, in terms of stability and resistance.

XRF results (Table 2) show that RHA consists of high silica oxide content (87.6%), and small percentages of potassium (2.87%), calcium (0.88%), iron (0.61%) and magnesium (0.33%) oxides. These oxides are important in pozzolanic materials, and which may vary depending on the combustion process^4^.

**Table 2:** RHA chemical composition.

Oxide	MgO	Al_2_O_3_	SiO_2_	P_2_O_5_	SO_3_	Cl	K_2_O	CaO	TiO_2_	MnO	Fe_2_O_3_	Others	Loss on ignition
Content (%)	0.33	0.09	87.6	0.48	0.22	0.09	2.87	0.88	0.01	0.36	0.61	0.05	6.41

**Figure 2:**
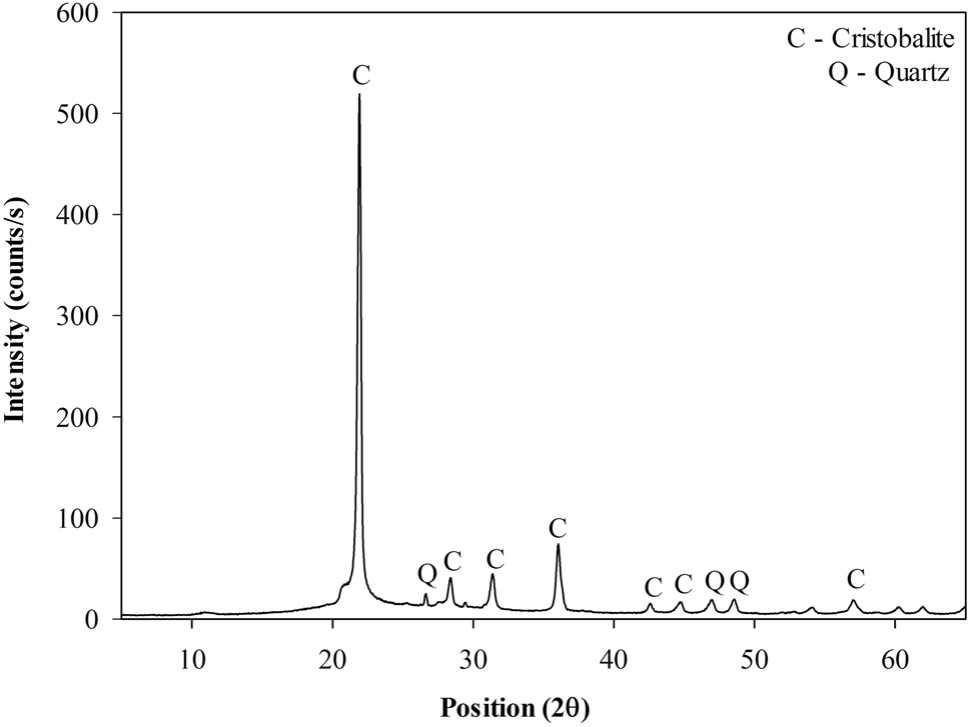
RHA mineralogical composition.

According to the Fratini method^27^ test result, RHA is a pozzolana with low reactivity, classified a class “N” pozzolana. This result is in agreement with the literature, e.g. RHA applied as a pozzolanic material in cement mixtures^7^. The RHA mineralogical composition (Fig. 2) is essentially cristobalite (SiO_2_) and quartz (SiO_2_), being considered a material with crystalline and amorphous phases.

**Figure 3:**
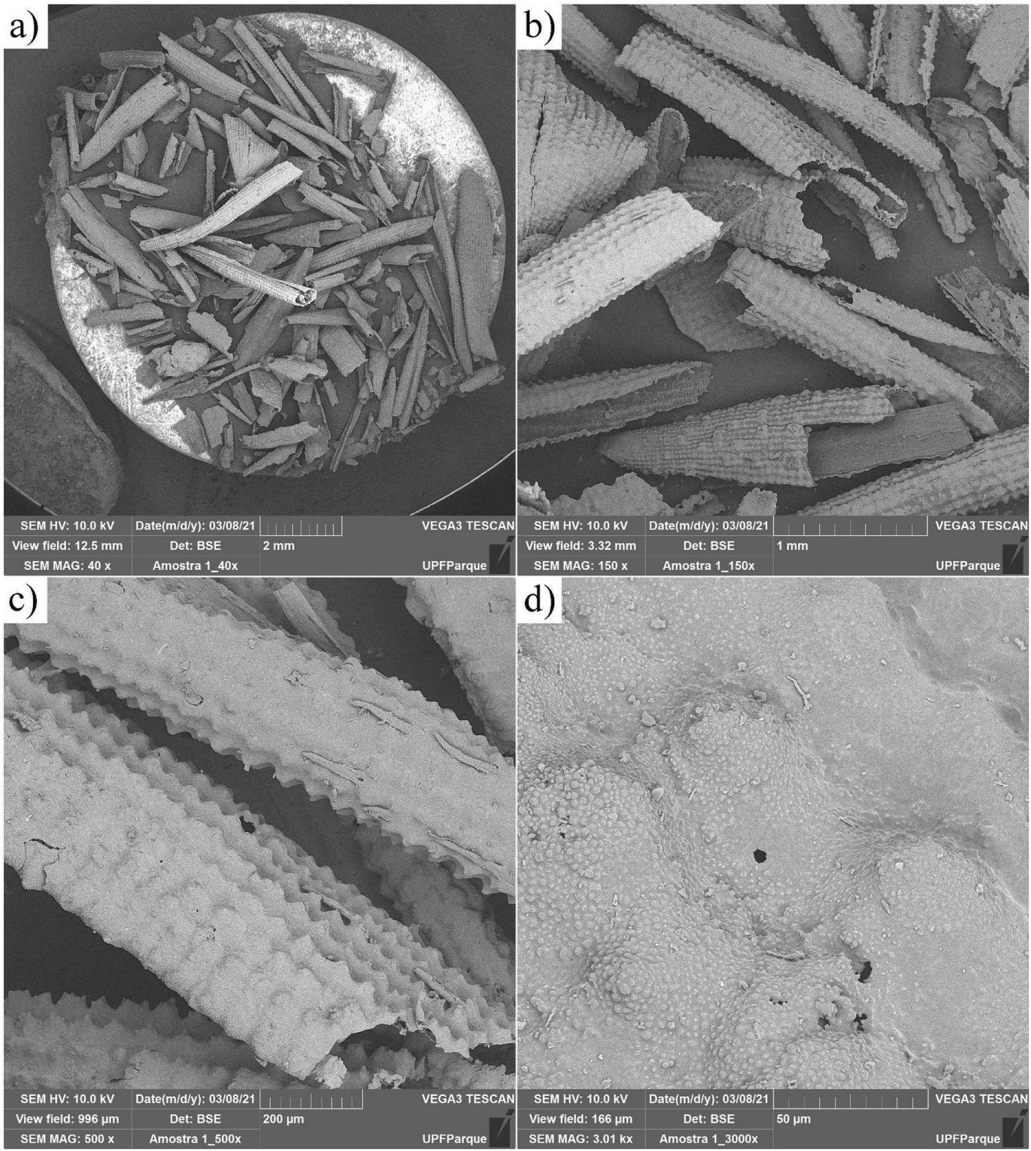
RHA morphology at ×40 (**a**), ×150 (**b**), ×500 (**c**) and ×3000 (**d**) magnification.

In terms of microstructure, Fig. 3 illustrates the morphology and microstructure of the RHA, using scanning electron microscopy (SEM) analysis. In addition, the presence of corn cob-shaped particles is verified (Fig. 3B), related to the organization of residue molecules in the backbone structure.

Leaching results (Table 3) show that the RHA does not present toxicity as no chemical element of the leached extract exceeded the limits established by Brazilian legislation (Annex F)^28^. However, the RHA presented solubilization of manganese, fluoride, and phenol compounds at concentrations higher than those indicated in Annex G^28^ (Table 4). Therefore, the RHA is classified as Class II A-Non-Hazardous Non-inert (e.g. it may present biodegradability, combustibility, or water-solubility properties)^28^. The concentration of solubilized manganese is justified by the significant presence of the element in the chemical composition of the RHA (0.36% MnO). Rice husks have phenolic acids in their composition^34^, which possibly explains the phenol solubilized in the RHA studied. Due to the rice husk^35^ and rice husk ash^36,37^ are efficient adsorbent materials for water defluoridation, it is believed that fluoride solubilized in the RHA comes from the adsorption of the element during the rice husk processing.

**Table 3:** Chemical composition of the leached and solubilized extract and respective limits according to NBR 10004^28^.

Element	Leaching tests		Solubilization tests	
	Results	Limits (mg/L)	Results	Limits (mg/L)
As	0.00	1.00	0.00	0.01
Ag	0.00	5.00	0.00	0.05
Ba	0.55	70.00	0.00	0.70
Cd	0.00	0.50	0.00	0.005
Pb	0.00	1.0	0.00	0.01
Cr	0.00	5.00	0.00	0.05
Fluoride	1.84	150.0	1.54	1.5
Hg	0.00	0.10	0.00	0.001
Se	0.00	1.00	0.00	0.01
Total phenol	–	–	< 0.04	0.001
Al	–	–	0.13	0.2
Fe	–	–	< 0.05	0.3
Mn	–	–	1.51	0.1
Na	–	–	9.65	200.0
Zn	–	–	0.06	5.0
Cu	–	–	0.00	2.0
Phenol	–	–	< 0.04	0.01
Nitrate	–	–	2.16	10.0
Chloride	–	–	95.55	250.0
Surfactant	–	–	< 0.20	0.5

**Table 4:** Factors and levels of experimental design.

Factor	Level − 1	Central	Level + 1
RHA (%)	2	21	40
Lime (%)	2	6	10
pH	2	4	6

The hydrated ESL presents 72.90% of calcium oxide and minerals in the form of portlandite (Ca(OH)_2_), calcite (CaCO_3_), and magnesium peroxide (MgO_2_)^21^. The CL is in its majority composed of 45.9% CaO and 23.6% MgO; constituted by portlandite, calcite, and magnesium-based minerals^3^. Additional information regarding hydrated ESL and CL properties can be found in Consoli et al.^21^ and Araújo et al.^3^, respectively.

The mineralogical composition of residual basalt soil consists of quartz (SiO_2_), hematite (Fe_2_O_3_), kaolinite (Al_2_(Si_2_O_5_)(OH)_4_) and anatase (TiO_2_)^38^. Geotechnical properties indicates a clayey silty soil according to grain size distribution^31^, with liquid limit (%) of 56, plastic limit (%) of 50 and plastic index (%) of 4^39^, and MH classification^40^, with high compressibility and medium plasticity.

### Reactive behavior in batch test

The reactive behavior, referring to the incorporation of RHA in a mixture with residual clayey soil and ESL or CL, was studied through batch tests. The determination of the RHA and both limes levels to be incorporated into the soil was carried out employing a central composite factorial experimental design, with the addition of face-centered axial points to investigate the non-linearity of the behavior, if necessary. The measured variable was the difference between the final and initial pH of the contaminating solution, and the control variables were initial pH (2 and 6), RHA content (2% and 40%) and lime content (2% and 10%). The experiments were conducted in 2 blocks for each lime. Table 4 presents factors and levels of experimental design.

The total content of RHA was limited to 40% to investigate the effects of carbonation of free lime in the results of strength and reactive behavior^41^. The pH range was adopted because soil changes under the effect of inorganic acidic attack, usually occur between pH ranges of 3–6^42^. Considering high acidity events, the literature recommends studying lower pH values, such as close to 1^22,38,43^. Batch tests were performed following standard D 4646-03^44^. The mass/solution ratio used at each experimental point was 1:20. The samples were shaken on an orbital shaker table, under 150 rpm, at a constant temperature of approximately 25 °C for a period of 24 h, to simulate an acidic attack on the mixtures. The response variable (reactivity) was calculated through the initial pH variation and after contact with the acidic solution.

### Physical–mechanical and reactive behavior of soil-RHA-lime mixtures

Based on preliminary results from the reactive behavior evaluation tests, 30% ash (in relation to the soil weight) was adopted to maximize the use of the waste in soil stabilization.

The ICL—initial consumption of lime test was used to determine the minimum lime content for pozzolanic reactions to occur in the ash-soil mixture. This method allows the evaluation of the lime increment ratio until the pH stabilization of the solution, which is composed of soil, ash, and distilled water^45^.

The particle size distribution of the mixtures and soil was performed using sieving and sedimentation^31^. Compaction tests were carried out for two mixtures, one with ESL (Mixture 1) and another with CL (Mixture 2) and a soil sample without ash or lime increment. The samples were prepared and compaction following the Brazilian standard^46,47^, adopting the normal Proctor energy.

The mechanical behavior of Mixtures 1 and 2 was evaluated using unconfined compression strength tests (UCS). The specimens were tested in a hydraulic press, according to the Brazilian standard^48^ after the curing time. The curing times studied were 7, 14, and 28 days, and the process took place in a humid chamber with a constant temperature of 23 ± 2 °C. Table 5 presents the soil- RHA-lime mixtures composition.

**Table 5:** Mixtures composition.

Mixture	ESL (%)	CL (%)	RHA (%)	Soil (%)
Mixture 1	6	–	30	64
Mixture 2	–	6	30	64

### Technological, and environmental characterization of the best performance mixture

The mixture with the best physical–mechanical and reactive performance was subjected to chemical and mineralogical characterization, after being subjected to rupture with 28 days of curing. The concentrations of chemical elements and the mineralogical composition of the mixtures were determined by the XRF and XRD techniques, respectively. The environmental performance of the best-performing mixture was also evaluated by analyzing the acidic contaminant (sulfuric acidic solution, pH 4) after contact with the mixture in a batch test, according to an adaptation of the D4646-03 standard^44^. After 24 h of testing, the sample was filtered and the chemical composition of the extract was analyzed by inductively coupled plasm atomic emission spectrometry (ICP-AES), and later compared with^49^, which provides information about limits and standards for soil quality and Annex G of NBR 10004^28^.

## Results and discussion

**Figure 4:**
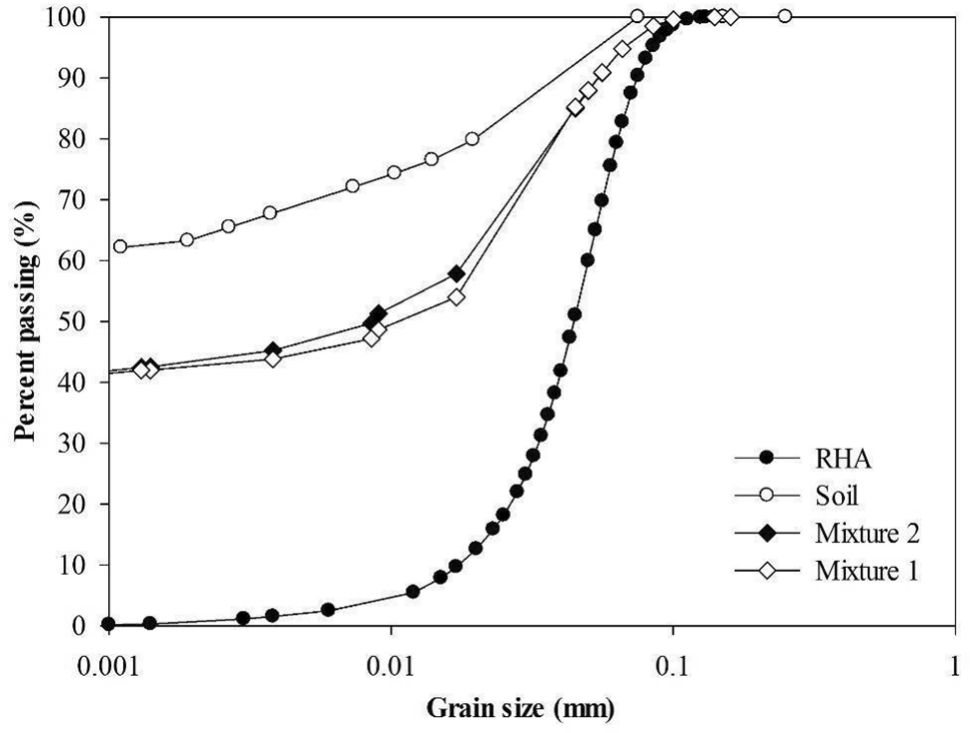
Particle size distribution of mixtures, compared to soil and RHA.

Figure 4 shows the distribution and particle size of mixtures 1 and 2, the soil, and RHA. The mixtures of the two materials with lime allowed the modification for a material with well-distributed particles of silt, clay, and sand, with similar behavior for the studied CL and ESL. Regarding the particle size distribution of the RHA, the material has an average diameter of 45 µm, with a uniform particle size distribution.

**Figure 5:**
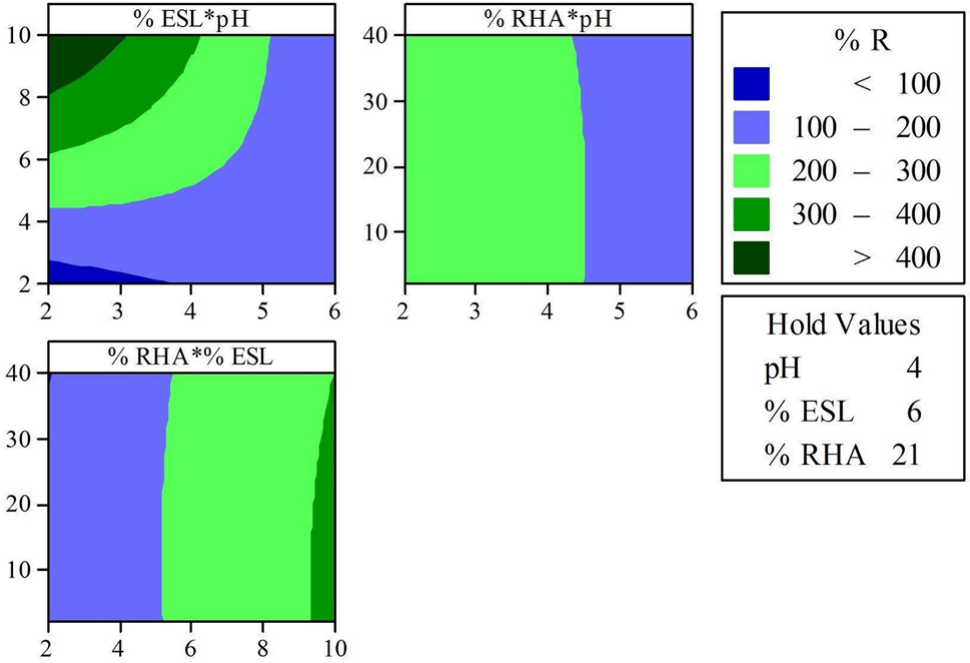
Contour surface of the experimental design of Mixture 1.

**Figure 6:**
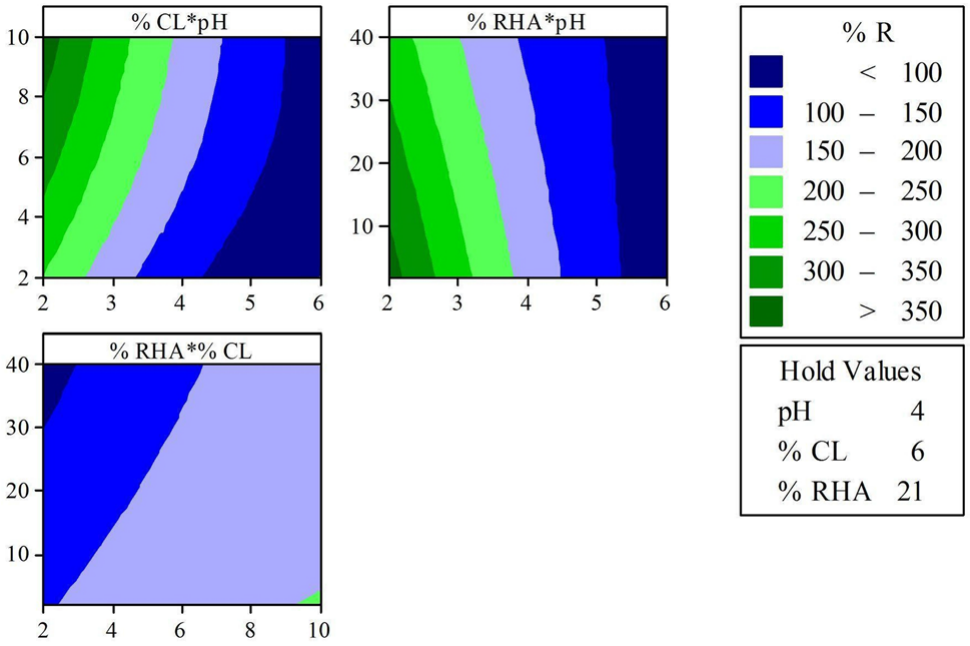
Contour surface of the experimental design of Mixture 2.

Figures 5 and 6 present the relationships of factors and response variables combined for Mixtures 1 and 2, using contour surfaces obtained after statistical analysis and modeling of the behavior of the response variables. After statistical analysis of variance with 95% significance, the variables that most significantly influenced UCS were the pH and the % CL and % ESL, followed by the % RHA, being only non-linear behavior was observed for pH in mixture 2.

For both mixtures, lower ash contents result in the best reactivities, also higher lime contents, and the most acidic pH range. The addition of lime to the soil is responsible for increasing the alkalinity of the mixtures and stabilizing it, through the combination and exchange of calcium ions^50^, which explains the directly proportional relationship between the increase in lime addition content and pH elevation.

The combination of CL contents from 4 to 8% with higher ash contents present reactivity of 150–200%. These results, when applied to leachates with a pH range of 3.5–4.5, corresponding to a pH elevation of 5–9, respectively. According to CONAMA 430^51^, effluents with a pH in the range of 5–9 can be released into water bodies without risk. Thus, the contact of the acidic contaminant with the soil mixture results in the reduction of environmental liabilities due to the percolation of high acidity leachates into groundwater and surface waters.

Mixture 1 with ESL showed a better ability to neutralize the contaminant's acidity, resulting in greater reactivity. These results show a significant reduction in the use of lime, as only 6% of ESL corresponds to higher reactivities than mixtures with 8% CL. This can be explained by the fact that eggshell lime has higher concentrations of calcium, reaching more than 72.90% of calcium oxide available for reaction, while CL has 43.56% CaO.

**Figure 7:**
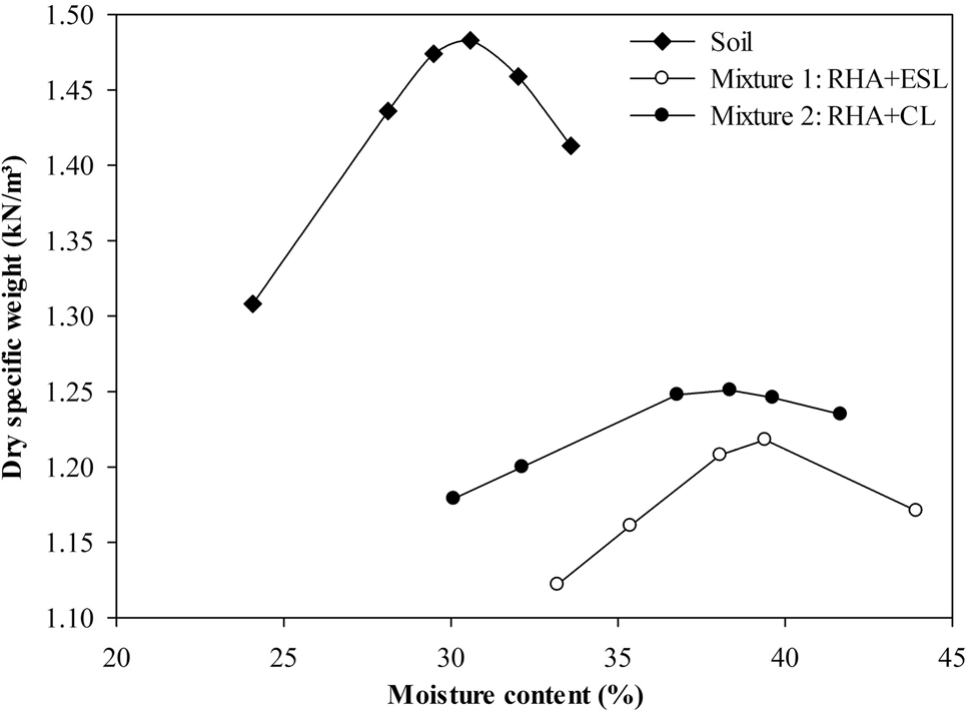
Compaction curves for Mixture 1 (ESL), Mixture 2 (CL), and soil.

The physico-mechanical behavior of the mixtures was evaluated with 6% lime (ESL or CL) (obtained using the ICL method)^45^ and 30% RHA, based on the reactivity results obtained previously. Figure 7 presents the soil compaction curves and the mixtures under study. The incorporation of RHA significantly increases soil moisture, previously with a maximum dry specific weight of 14.5 kN/m^3^ and an optimal moisture content of 30%. The mixtures with the addition of CL and ESL behaved similarly, however, the mixtures with ESL had lower densities and higher humidity when compared to the mixtures with the addition of CL.

The addition of RHA to the soil significantly affects the parameters of moisture content and dry unit weight (γd), being beneficial for fine-grained soils, as it presents an improvement of behavior of soil without compaction, decreasing the dry specific weight and increasing the optimum moisture content. The specimens submitted to the UCS test were cast under the conditions presented with maximum dry specific weight and optimum moisture content, being 12.5 kN/m^3^ and 36.1% for the mixture with commercial lime (CL), and 12.1 kN/m^3^ and 39.1% for the mixture with eggshell lime (ESL).

**Figure 8:**
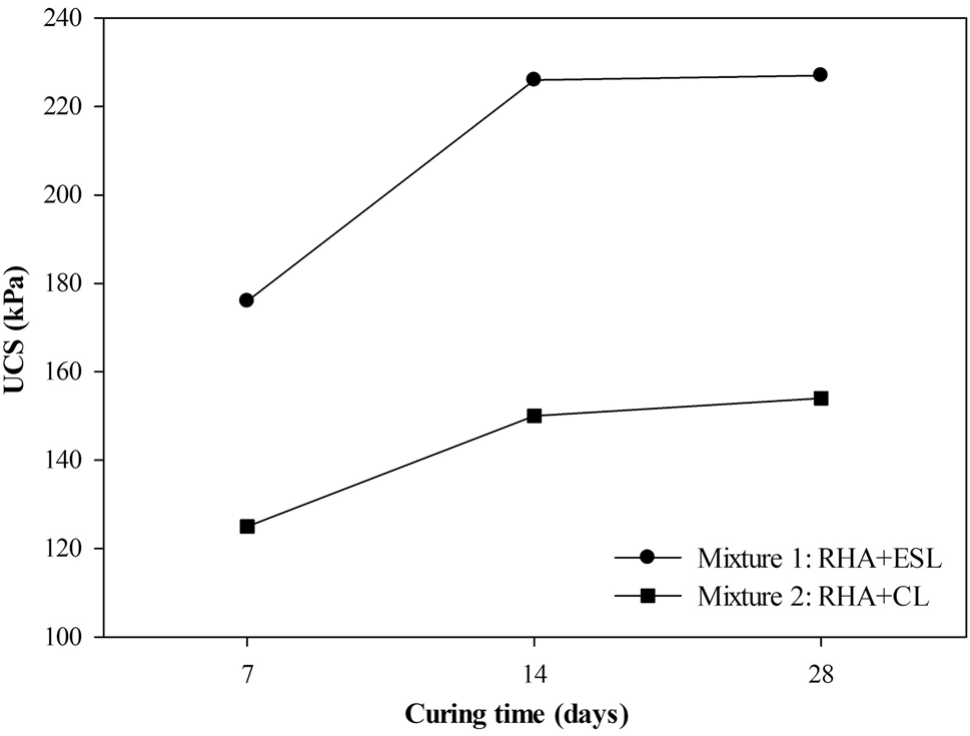
Unconfined compression strength at 7, 14, and 28 days of curing the mixtures.

Figure 8 shows the unconfined compression tests of both mixtures for curing times of 7, 14, and 28 days. Mixture 1 shows greater strength than Mixture 2 due to high calcium oxide content (72.9%) from ESL available for pozzolanic reactions. The mixtures with 28 days of curing did not give high strengths, stabilizing after 14 days (Fig. 8). However, the results obtained allow the mixtures application, with the minimum strength being 200 kPa (e.g. impermeable barriers of landfills)^52^, allowing the application of mixtures with ESL with 14 days of curing (226 kPa).

The UCS results are explained by the crystalline silica predominance compared to the amount of amorphous silica in the RHA. Furthermore, RHA has a substantial concentration of silica oxide and low aluminum, which decreases the formation of aluminosilicates, reducing the mixture's strength^53^. RHA mixtures pre-treated by grinding can produce better results, the smaller particles fill the voids and help the formation of a denser and stronger specimen. Despite this, RHA was used in natural conditions, allowing it to be applied without pretreatment costs.

Table 6 presents the environmental performance results of Mixture 1. It is possible to notice that only two chemical elements were detected in the acidic contaminant extract: aluminum (Al) and sodium (Na). Bearing in mind that the origin of these elements is the crude RHA, as they are the elements present in higher concentrations in the solubilized extract of the RHA. The limits of national and international water quality standards CONAMA 460^49^, Dutch list^54^, EPA^55^ and NBR 10004-Annex G^28^ were not exceeded for both metals in the Mixture 1.

**Table 6:** Metal concentrations of mixture 1 after acidic attack.

Element	Concentration (mg/L)	NBR 10,004 (Annex G) Limit^a^	Solubilized extract RHA (mg/L)	CONAMA 460 limit^b^	Dutch list limit^c^	EPA limit^d^
Ag	*	0.05	*	0.05	–	–
Al	0.020	0.200	0.132	3.50	–	–
As	*	0.01	*	0.01	0.01	0.01
Ba	*	0.70	*	0.70	0.05	2
Cd	*	0.005	*	0.005	0.0004	0.005
Cr	*	0.05	*	0.05	0.001	0.1
Cu	*	2	*	2	0.015	1.3
Fe	*	0.3	< 0.006	2.45	–	–
Hg	*	0.001	*	0.001	0.00005	0.002
Mn	*	0.1	1.5	0.4	–	–
Na	1.32	200	9.6	–	–	–
Pb	*	0.01	*	0.01	0.015	0.015
Se	*	0.01	*	0.01	–	0.05
Zn	*	5	0.06	1.05	0.065	-

*Below detection limit; ^a^solubilized contaminant contents; ^b^guiding values of groundwater and drinking water; ^c^target values of groundwater; ^d^maximum contaminant levels—national primary drinking water regulations.

The addition of eggshell lime in the soil provides a reduction in toxicity, contributing to the inertization of RHA in the mixture. This was also observed by Soares, Quina and Quinta-Ferreira^56^, who studied the incorporation of ESL in soil, and observed the immobilization of heavy metals such as lead (Pb) and zinc (Zn), with acidic conditions favorable to Zn retention, as also observed in the present study with the acidic attack under pH 4.

XRF results (Table 7) show that Mixture 1 is composed mainly of silica (40.3%), iron (21.9%), aluminum (18.0%), and calcium oxides originated from the raw materials used in the mixture, i.e., clayey soil, RHA and ESL, respectively.

**Table 7:** Chemical composition of Mixture 1.

Oxide (%)	MgO	Al_2_O_3_	SiO_2_	P_2_O_5_	SO_3_	Cl	K_2_O	CaO	TiO_2_	MnO	Fe_2_O_3_	Others	Loss on ignition
Mixture 1	0.27	18.00	40.30	0.20	0.24	0.03	0.67	3.72	2.95	0.22	21.90	0.20	11.30
RHA	0.33	0.09	87.6	0.48	0.22	0.09	2.87	0.88	0.01	0.36	0.61	0.05	6.41

**Figure 9:**
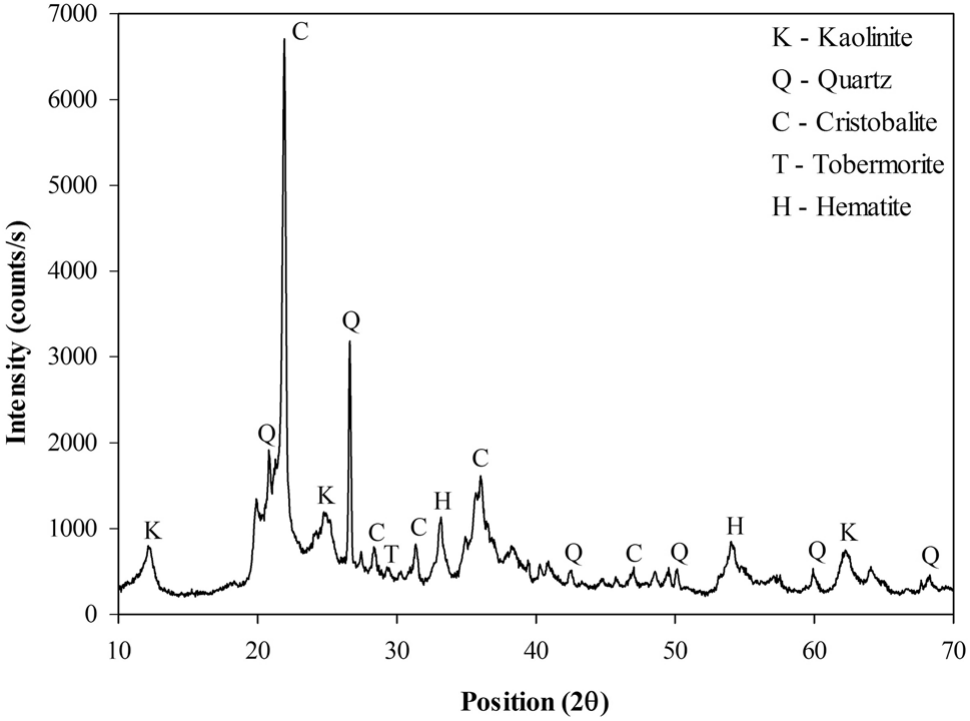
Mineralogical composition of the Mixture 1 after 28 days of cure time.

The XRD (Fig. 9) indicates the presence of kaolinite (Al_2_(Si_2_O_5_)(OH)_4_), quartz (SiO_2_), cristobalite (SiO_2_), tobermorite (Ca_5_Si_6_O1_6_(OH)_2_·4H_2_O) and hematite (Fe_2_O_3_). Kaolinite, hematite, quartz and cristobalite originated from the soil and the RHA, respectively. The mineral tobermorite, which resembles the structure of hydrated calcium silicate gel (C–S–H), was observed in poorly crystalline phases. Also, portlandite (the main constituent of ESL) was not identified in the diffractogram, indicating that this mineral was consumed on the pozzolanic reactions.

## Conclusion

Based on the findings of this study, the following conclusions were drawn:

- The incorporation of the RHA-ESL binder to a clayey residual soil allowed a satisfactory mixture for the improvement of the mechanical and reactive behavior, promoting the partial encapsulation of metals present in the rice husk ash;
- 30% RHA in the soil with 6% ESL represented the mixture with the best reactive behavior for the neutralization of acidic contaminants in the soil, as it raised the pH and prevented the solubilization of toxic elements;
- The addition of ESL presented satisfactory environmental performance considering the applicable regulations;
- ESL showed to be a more efficient binder than CL in terms of mechanical strength and reactivity;
- XRF results show that the soil-RHA-ESL mixture is composed majorly by silica (40.3%), iron (21.9%) and aluminum (18.0%) oxides;
- XRD of soil-RHA-ESL mixture indicates the presence of kaolinite, quartz, cristobalite, tobermorite and hematite. A C–S–H gel is observed in poorly crystalline phases through the XRD pattern;
- Because of the maximum UCS achieved after 14 and 28 curing days, an engineering application of the soil-RHA-ESL mixture could be foreseen in impermeable barriers of landfills.

## Data Availability

The datasets used and/or analyzed during the current study are available from the corresponding author on reasonable request.
